# Application of Nuclear Volume Measurements to Comprehend the Cell Cycle in Root-Knot Nematode-Induced Giant Cells

**DOI:** 10.3389/fpls.2017.00961

**Published:** 2017-06-12

**Authors:** José Dijair Antonino de Souza Junior, Olivier Pierre, Roberta R. Coelho, Maria F. Grossi-de-Sa, Gilbert Engler, Janice de Almeida Engler

**Affiliations:** ^1^Institut National de la Recherche Agronomique, Université Côte d’Azur, Centre National de la Recherche Scientifique, Institut Sophia AgrobiotechSophia-Antipolis, France; ^2^Laboratório de Interação Molecular Planta-Praga, Embrapa Recursos Genéticos e BiotecnologiaBrasília, Brazil

**Keywords:** nuclei, giant cells, nematode feeding sites, galls, root-knot nematodes

## Abstract

Root-knot nematodes induce galls that contain giant-feeding cells harboring multiple enlarged nuclei within the roots of host plants. It is recognized that the cell cycle plays an essential role in the set-up of a peculiar nuclear organization that seemingly steers nematode feeding site induction and development. Functional studies of a large set of cell cycle genes in transgenic lines of the model host *Arabidopsis thaliana* have contributed to better understand the role of the cell cycle components and their implication in the establishment of functional galls. Mitotic activity mainly occurs during the initial stages of gall development and is followed by an intense endoreduplication phase imperative to produce giant-feeding cells, essential to form vigorous galls. Transgenic lines overexpressing particular cell cycle genes can provoke severe nuclei phenotype changes mainly at later stages of feeding site development. This can result in chaotic nuclear phenotypes affecting their volume. These aberrant nuclear organizations are hampering gall development and nematode maturation. Herein we report on two nuclear volume assessment methods which provide information on the complex changes occurring in nuclei during giant cell development. Although we observed that the data obtained with AMIRA tend to be more detailed than Volumest (Image J), both approaches proved to be highly versatile, allowing to access 3D morphological changes in nuclei of complex tissues and organs. The protocol presented here is based on standard confocal optical sectioning and 3-D image analysis and can be applied to study any volume and shape of cellular organelles in various complex biological specimens. Our results suggest that an increase in giant cell nuclear volume is not solely linked to increasing ploidy levels, but might result from the accumulation of mitotic defects.

## Introduction

Plant-parasitic nematodes are a major threat to many plant cultures worldwide, and root-knot nematodes (RKN) are responsible for extensive economic losses caused by phytonematodes (Chitwood, 2003). Cyst nematodes (CN) are also a major group of plant-parasitic nematodes causing great economic losses worldwide (Turner and Subbotin, 2013). The major genus that belongs to CN are *Heterodera* and *Globodera*.

**FIGURE 1:**
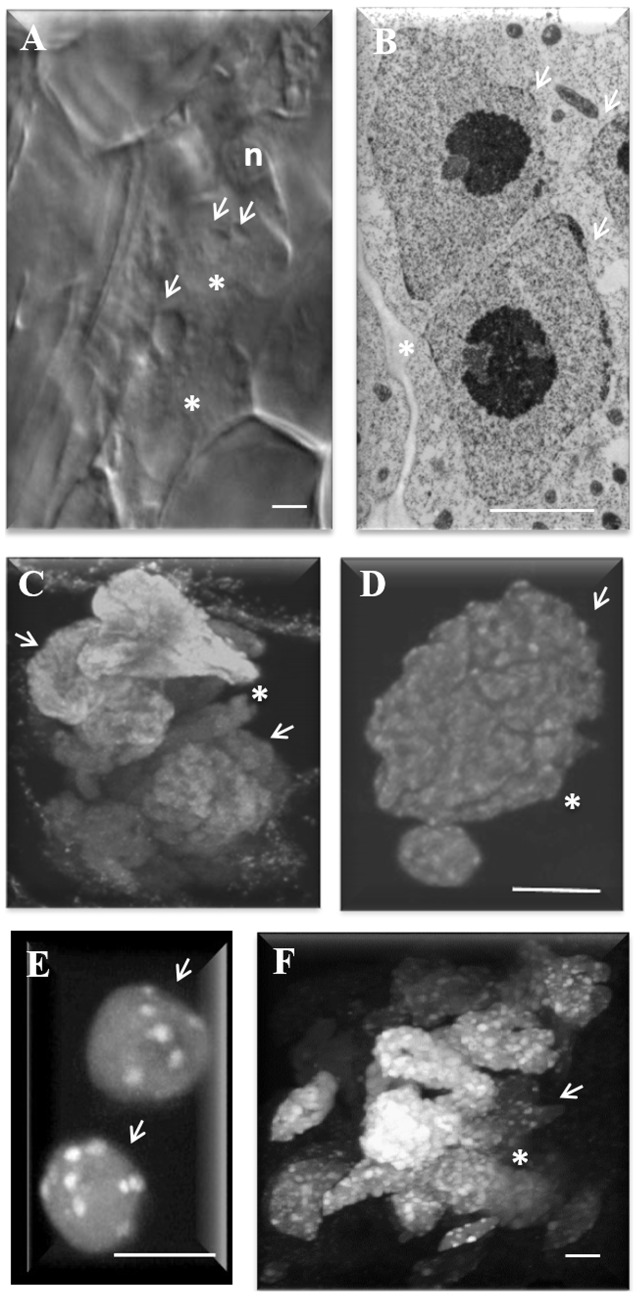
Nuclear morphology in nematode-induced giant cells (GC) in Arabidopsis roots. **(A)** Differential interference contrast image of GC 3 days after inoculation (DAI) undergoing initial nuclear divisions. **(B)** Transmission electron microscopy image of a recently divided nucleus. **(C,D)** Maximum projection of confocal images of DAPI (4,6-diamidino-2-phenylindole) stained nuclei showing, **(C)** convoluted nuclei in GC, and **(D)** the groovy surface of a GC nucleus. **(E)** DAPI-stained wild-type root nuclei showing up to 10 chromocenters (white dots) and **(F)** GC nuclei 20 DAI showing numerous chromocenters (white dots). White arrows point to nuclei. ^∗^, GC; n, nematodes. Bars = 5 μm.

Feeding sites induced by RKN are composed of outsized feeding cells named “giant cells” (GCs) which hold a dense cytoplasm filled with organelles and enlarged nuclei, representative of high metabolic activity. These giant-feeding cells are surrounded by mitotically active vascular tissue cells characterized by asymmetric cell wall positioning. This peculiar division pattern gives rise to a multi-layered shell consisting of small cells neighboring the GCs. This finally outlines the typical knot shape in susceptible roots called a “gall.” On the other hand, CN initiate a syncytium through elongation of a single initial feeding cell, accompanied by division of neighboring cells which will fuse as syncytia expands (Golinowski et al., 1997). Unlike RKN-induced GCs, the multinucleated state of CN-induced syncytia is achieved by cell wall dissolution of these neighboring cells (Rodiuc et al., 2014; Bohlmann, 2015) rather than by mitotic activity of the syncytia itself. Moreover, first steps of gall formation involve the redifferentiation of vascular cells into binucleate GCs which become multinucleate via succeeding nuclear divisions (**Figure 1A**). Nematode-induced GCs undergo multiple and often synchronous acytokinetic mitotic events (**Figure 1B**) (Jones and Payne, 1978; Banora et al., 2011; Vieira et al., 2013).

Giant cells of Arabidopsis hold up to 60 nuclei which become particularly enlarged, often grouped, presenting peculiar shapes with variable sizes and irregular surfaces (**Figures 1C,D**). Cytogenetic studies revealed that giant cell nuclei are polyploid or aneuploid (Wiggers et al., 1990; Starr, 1993). Confocal images of three-dimensional reconstruction of gall nuclei at different time points after nematode infection confirmed variable nuclei morphology and size within GCs and the multiplicity of often synchronous mitotic events (Vieira et al., 2012). DAPI stained nuclei in diploid root cells of Arabidopsis present approximately 10 chromocenters (**Figure 1E**) while giant cell nuclei present profuse chromocenters differently sized suggestive of nuclei polyploidization (**Figure 1F**). Chromocenters are densely stained heterochromatic regions matching to centromeres and nuclear organizing regions (Fransz et al., 2002). Endoreduplication is recognized to affect chromatin dynamics and the number and size of these centromeric heterochromatin domains in interphase nuclei can be indicative of endocycle activity. This type of DNA amplification is the major process involved in the increase of ploidy in GCs (de Almeida Engler et al., 1999, 2012; de Almeida Engler and Gheysen, 2013). The latter is illustrated by the increase of DNA replication cycles occurring in GCs as shown by ^3^H-thymidine incorporation studies (Rubinstein and Owens, 1964; Rohde and McClure, 1975; de Almeida Engler et al., 1999, 2015). Functional studies on cell cycle genes (e.g., *AtCCS52B* gene family) also support the implication of the endocycle in giant cell development (de Almeida Engler et al., 2012).

Up to now, there is no evidence that endomitosis may take place in GCs considering that chromosome condensation has never been observed within the nuclear membrane. Therefore, it is most likely that polyploidy in GCs is mainly achieved via the endocycle by raising global cell cycle gene expression levels within GCs. This might contribute to the overall increase in metabolic activity required for GC growth and an increased cytoplasm content essential to provide sufficient resources for nematode feeding (de Almeida Engler et al., 2012, 2015; de Almeida Engler and Gheysen, 2013).

In many somatic plant cells, DNA C-values continue to increase by endoreduplication in which nuclear DNA replication is irreversibly uncoupled from mitosis. The early literature describes that high mitotic activity in GCs may result in chromosome fusion of adjacent metaphases unequal distribution to the daughter nuclei or to incomplete mitotic events (Wiggers et al., 1990; de Almeida Engler et al., 2004). DNA content in GCs can further increase for at least 2 weeks after mitosis has ceased and flow cytometric ploidy measurements confirmed the occurrence of extensive endoreduplication in GCs (Vieira et al., 2013, 2014).

Mitotic defects and consequently nuclear phenotype changes are often observed to occur in GCs ectopically expressing cell cycle genes. Recently, it has been reported that severe morphological changes like convoluted and apparently connected nuclei are formed in lines overexpressing members of the *KRP* gene family known as inhibitors of cyclin-dependent kinases (CDKs) and their regulatory subunits (CYCs) [see Vieira and de Almeida Engler in same issue and (Vieira et al., 2012, 2013, 2014; Coelho et al., 2017)]. The appearance of complex nuclear phenotypes prompted us to apply other techniques besides flow cytometry to get a more accurate picture of the relation nuclear volume/shape and ploidy. Indeed, although it is generally accepted that endoreduplication leads to an increase in nuclear volume, the outsized and highly irregular nuclear shapes observed in the lines studied here, suggest that other mechanisms might be responsible for the nuclear phenotypes observed. Therefore, we opted to accurately measure nuclear volumes in GCs induced by the RKN *Meloidogyne incognita* using thick slices of nematode infection sites and confocal microscopy imaging. Optical sections of galls were subjected to nuclear volume measurements that were performed by two independent softwares; the public domain Volumest plugin from ImageJ and the AMIRA 3D software for visualization and analysis. Comparative volumetric measurements of GC and neighboring cells here named non-giant cells (NGC) nuclei present in wild-type (Col-0) and *KRP3^OE^* and *KRP5^OE^* Arabidopsis transgenic lines (Coelho et al., 2017) were performed and results are presented herein.

## Materials and Methods

### Nematode Infection

Seeds from wild-type *Arabidopsis thaliana* Columbia (Col-0) and transgenic lines *35S::KRP3-GFP* and *35S::GFP-KRP5* (designated *KRP3^OE^* and *KRP5^OE^* hereafter) were sown on cups containing a sterile mix of sand and soil (2:1). After cold treatment (2 days at 4°C) in the dark, seeds were kept in a growth chamber with a 12-h light:12-h dark photoperiod at 21°C. Fourteen-day-old seedlings were then individually placed in a sand/soil mixture and 1 week later, roots were inoculated with 200 freshly hatched, second-stage juveniles (J2s) of *M. incognita*. Infections were performed in two independent experiments for both transgenic and wild-type lines. Galls were subsequently collected at 14 and 21 days after inoculation (DAI).

### Gall Sample Preparation for Confocal Analysis

**FIGURE 2:**
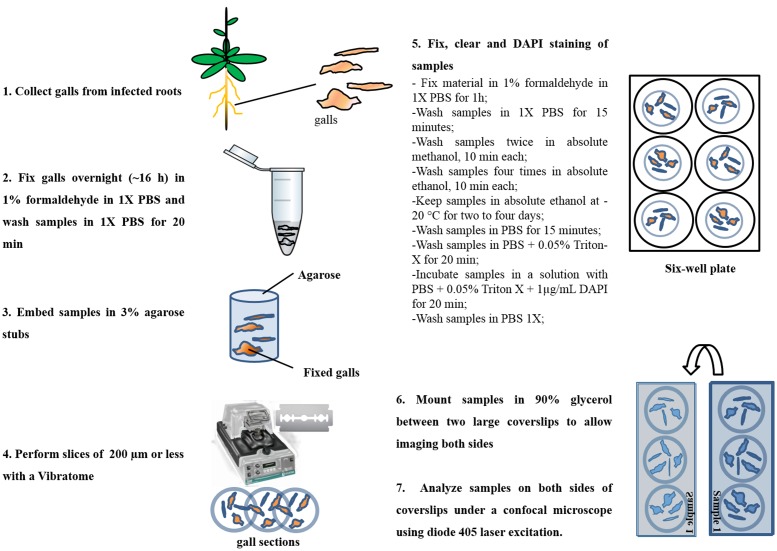
Flowchart of the procedure to prepare thick slice using a Vibratome and nuclei staining. *Meloidogyne incognita*-induced galls 14 and 21 DAI were prepared for confocal imaging and posterior nuclei morphology observation and volumetric measurements by ImageJ plug-in, Volumest, and AMIRA softwares.

Nuclear analyses of cleared thick gall slices (named vibroslices thereafter) made with a Vibratome (Vibratome 100 plus, United States) were basically treated as described in Vieira et al. (2012) with minor modification as outlined in **Figure 2**. Nematode-infected roots from 14 to 21 DAI were fixed in phosphate-buffered saline (PBS), pH 7.2, containing 1% formaldehyde (PBSF) for 16 h at 4°C. Samples were embedded in liquid 3% agarose (Thermo-Fischer, United States) in small plastic stubs. When solidified, agarose blocks were removed and mounted on the sample holder of the Vibratome, slices of 150–200 μm were generated floating on distilled water. Vibroslices containing gall tissue were fished and subsequently fixed in PBSF for 1 h at RT. Then samples were dehydrated twice with absolute methanol and four times with absolute ethanol (both stored at 4°C) 10 min each step. Vibroslices were stored at -20°C for 2–4 days to allow tissue clearing. Subsequently, they were washed twice for 15 min with PBS. Vibrosliced galls were then incubated in a solution containing PBS, 0.05% Triton-X 100 (Sigma–Aldrich, United States), and 1 μg/ml DAPI (Thermo-Fischer, United States) for 20 min. Finally, the samples were washed in PBS for 5 min and mounted with care to avoid compression in 90% glycerol between two coverslips to allow double side confocal imaging.

### Confocal Imaging of DAPI Stained Nuclei in Cleared Gall Slices

Cleared vibrosliced galls 7 and 21 DAI from wild-type Col-0, 14 and 21 DAI *KRP3^OE^* and *KRP5^OE^* transgenic lines (Coelho et al., 2017) were imaged using a Leica SP8 confocal microscope with objective lens HC PL APO CS1 40X/1.30 OIL. DAPI was excited using a diode 405 nm laser and fluorescence was collected between 431 and 532 nm. All samples were scanned at 400Hz and 16X line averaged for high-quality image recording. Z-stacks generated from approx. around 100–150 images per sample with a 0.44 μm optical slice thickness were a source for volumetric measurements and maximum brightness projections were used for illustrative figures.

### Volumetric Measurement of Giant and Non-giant Cells Nuclei

Image stacks of DAPI-stained galls were analyzed with the plugin Volumest^1^ from the public domain ImageJ software (NIH, Bethesda, MD, United States). Volumest allows volume estimation using confocal data sets. Before estimating the volume of GCs nuclei, 3D projections of each image stack were made with ImageJ, to facilitate nuclei mapping. Then, original z-stacks were prepared as required for Volumest analysis. Volume measurements were performed on a selection of nuclei with bright DAPI staining as identified from maximum projection images. This selection was necessary to avoid measuring background noise. Well defined nuclei were subsequently measured using the cumulative nuclei volume tool and recorded as shown in the Supplementary File 1. Individual nuclear volumes are depicted in Supplementary File 1 and the total volume of GC or NGC nuclei is divided by the number of measured nuclei resulting in the average nuclear volume per gall.

To simplify the measuring procedure we assessed the nuclei volume from a single Col-0 image stack at different manners. First, the nuclear volumes were computed by summing the volumes of each individual image in the stack. Then volumes were measured by skipping one, two, three or four images in the same sequence taking into account the slice thickness. All measurements were carried out on at least 400 GC nuclei with a minimum of 22 GCs for each time point for each line. Also, the volume of NGC was measured with more than 100 per time point for each line.

All data generated were analyzed statistically by Sigma Plot version 12 (Systat Software, Inc., United States) using one-way analysis of variance (ANOVA). The Shapiro–Wilk test was used for normality, the Levene test for homogeneity of variance and Tukey’s test to compare the means. Different letters between groups indicate a significant difference at *P* < 0.05.

### Confocal Image Stacks Processed with the AMIRA 3D Visualization and Analysis Software

In order to confirm the reliability of the data obtained using the ImageJ Volumest software, the same dataset was also analyzed with the AMIRA software package (FEI, Hillsboro, OR, United States). For the latter, nuclei were segmented according to standard procedures and correspondingly volume rendered. Briefly, to enhance the signal to noise ratio while preserving edges, images were first filtered with a 2D-median rolling ball, then nuclei were segmented by applying a white Top-Hat filter. Sorted out nuclei from gall sections were then filled to avoid holes within the selection. GC and NGC nuclei were individually labeled via the AMIRA segmentation tool and analyzed for morphologic traits.

## Results

### An Easy Protocol to Image Nuclei from Giant Cells Using Confocal Microscopy

**FIGURE 3:**
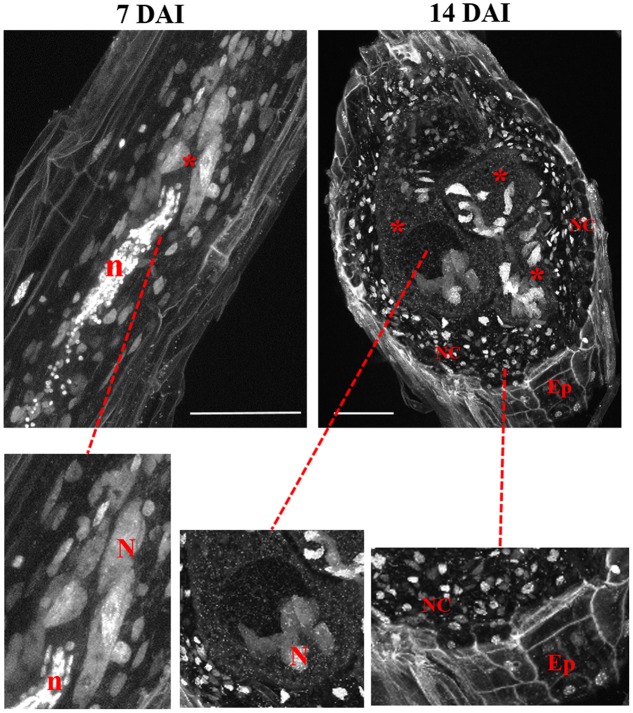
Maximum brightness projections of confocal images recorded of *M. incognita*-induced galls in Arabidopsis roots at 7 and 14 DAI. Both micrographs are thick sections allowing the visualization of DAPI-stained nuclei and DNA containing organelles like mitochondria and plastids in GC, neighboring cells, and epidermis. Details show nuclei in GC, neighboring cells, and epidermis. ^∗^, GC; NC, neighboring cells; N, nucleus; Ep, epidermis, n, nematode. Bars = 50 μm.

The protocol here described (summarized in **Figure 2**) is essentially based on a method developed for the microscopic analysis of whole mounts of RKN infected roots (Vieira et al., 2012). Here we used thick slices (vibroslices of 150–200 μm) made with a Vibratome considering that sliced galls are more accessible for staining with dyes like DAPI and for confocal imaging. This resulted in a better contrast and image quality even when using relatively thick slices (**Figure 3**). Therefore, this procedure allows a detailed analysis of GC nuclei shape, structure, volume, and spatial distribution. Other modifications include shorter sample preparation time, the use of DAPI instead of propidium iodide (PI); the former having a higher specificity for DNA. Samples were finally mounted between two coverslips (**Figure 2**) to allow imaging both sides of the gall sections.

Cleared vibroslices of DAPI stained galls 7 DAI, were used to image the initial stages of giant cell development allowing to observe enlarged nuclei close to the nematode head (**Figure 3**). Nematode nuclei are also clearly visible in DAPI stained samples being round shaped and smaller than GC nuclei. In galls (14 DAI) we could observe enlarged and often grouped nuclei in expanding GCs as well as individual nuclei in neighboring cells (**Figure 3**). Dark areas correspond to vacuoles while the very small DAPI stained fluorescent dots within the GCs are plastids and/or mitochondria. Cell walls are weakly stained due to background DAPI staining delimiting the living cells from dead xylem tissue.

### Measuring Volumes of Giant Cell Nuclei Using Volumest

**FIGURE 4:**
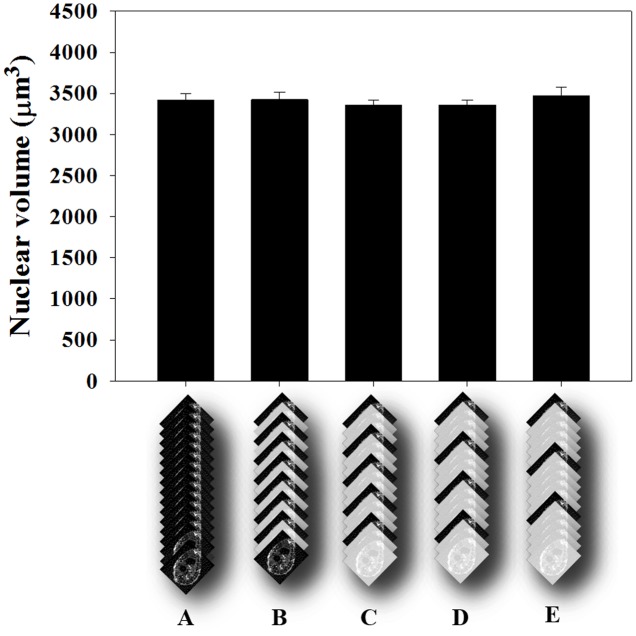
Average volume estimation of nuclei in GC of Arabidopsis roots. **(A)** Confocal optical sections covering 60 μm depth were measured using the ImageJ plug-in, Volumest. The z-stack contained 130 images where each nuclei volume was recorded. **(A)** Result obtained for each section measured. **(B)** Measuring one and skipping the next section. **(C)** Measuring one and skipping every two next sections. **(D)** Measuring one and skipping the next three sections. **(E)** Measuring one and skipping the next four sections. No statistical difference was detected after ANOVA test (*p* > 0.05). Subsequent measurements were then performed like in **D**.

Herein, we describe a methodology to measure nuclear volumes from plant root cells, especially from GCs induced by *M. incognita*. Therefore, we used confocal image stacks to assess the GC nuclear volume using the ImageJ plugin, Volumest software (here on mentioned as Volumest). This plugin allows estimating volumes from confocal acquired z-stack. In this way, we were able to estimate the GC nuclear volume by measuring the nuclear area from different gall confocal optical sections within a z-stack (Supplementary File 1). As a result, we assessed the volume of each individual nucleus. First, we performed a z-projection of the dataset to map nuclei within galls. Then, we measured the cross section area for each image optical section of the stack to estimate the total nuclear volume. Finally, we divided the total volume by the number of nuclei measured to determine the average nuclear volume. Since evaluating the confocal sections in a complete stack is time-consuming, we simplified our measuring procedure by assessing the GC nuclei volume from a wild-type Col-0 image stack by skipping one up to four images of the stack recordings (**Figure 4**). No differences were observed in the final nuclear volume when measuring only 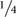 of the optical sections taking into account four times bigger optical slice thickness. Therefore, all volumetric measurements of GC nuclei were performed by measuring one and skipping the next three confocal slices.

### Applying the Volumest Method to Investigate the Effect of the Ectopic KRP3 and KRP5 Expression on Giant Cell Nuclear Volume

**FIGURE 5:**
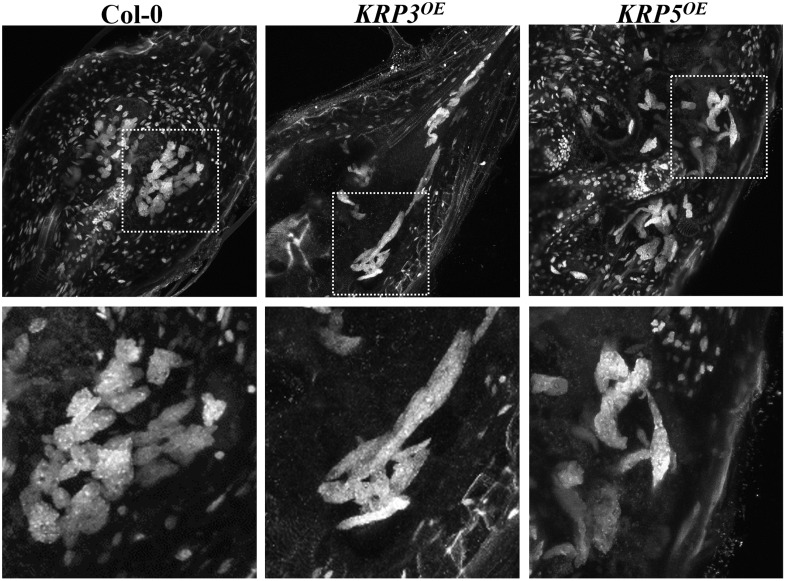
Ectopic KRP3 and KRP5 expression cause major nuclear morphology changes in GC induced by the root-knot nematode *M. incognita*. DAPI-stained samples clearly illustrate the morphological differences of the nuclei in GC of galls 21 DAI in wild-type compared to *KRP3^OE^* and *KRP5^OE^* lines. While wild-type nuclei are predominantly amoeboid in shape, nuclei in the *KRP3^OE^* and *KRP5^OE^* lines are elongated and apparently connected.

**FIGURE 6:**
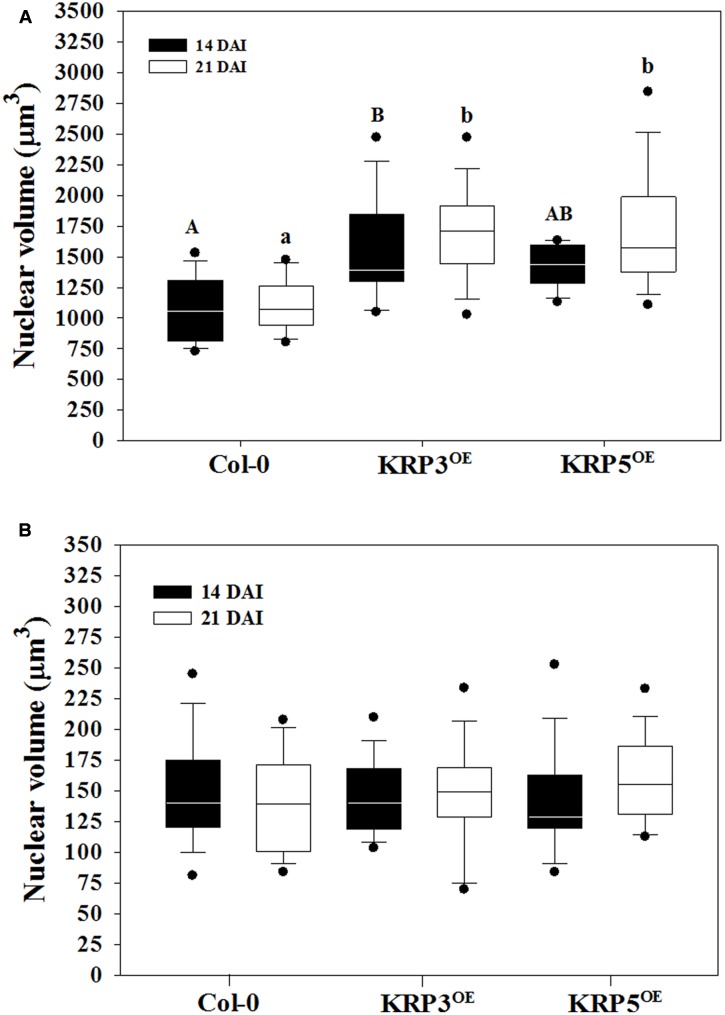
Average volume of nuclei in GC 14 and 21 DAI and non-giant cells (NGC) of wild-type (Col-0), *KRP3^OE^*, and *KRP5^OE^* lines. Measurements were performed using the ImageJ plug-in Volumest. **(A)** GC nuclear volume indicated with a capital letter for 14 DAI and small letter for 21 DAI mean statistical differences after ANOVA and Tukey tests (*p* < 0.05). Assays were performed in duplicates. There were no statistical differences between samples of 14 and 21 DAI within the same line. **(B)** In NGC, nuclear volume measurements no significant differences were observed between the three lines studied after ANOVA test.

To acquire information other than nuclear size and morphology (Coelho et al., 2017), we estimated nuclear volumes of Arabidopsis plants overexpressing the cell cycle inhibitors KRP3 and KRP5 compared to wild-type applying the Volumest software. We already observed that nuclei from GCs induced in plants overexpressing KRP3 and KRP5 have a peculiar morphology, presenting elongated and apparently connected GC nuclei in contrast with nuclei in wild-type GC that are mostly amoeboid-shaped and convoluted (**Figures 1**, **5**) (Coelho et al., 2017). In order to evaluate whether nuclear morphology in GCs upon ectopic KRP3 and KRP5 expression featured an effect on nuclei volume of both GCs and NGCs, we performed volumetric nuclear measurements in gall cells 14 and 21 DAI. Our method shows that nuclear volume of GCs in galls of 14 and 21 DAI was not significantly different (**Figure 6**). However, the nuclear volume from *KRP3^OE^* (14 and 21 DAI) and *KRP5^OE^* (21 DAI) was statistically larger than those of the wild-type (**Figure 6A**). No differences were observed between the nuclear volumes of NGCs at any time point (**Figure 6B**).

### Applying the AMIRA Method to Investigate the Effect of the Ectopic KRP3 and KRP5 Expression on Giant Cell and Non-giant Cell Nuclear Volume

**FIGURE 7:**
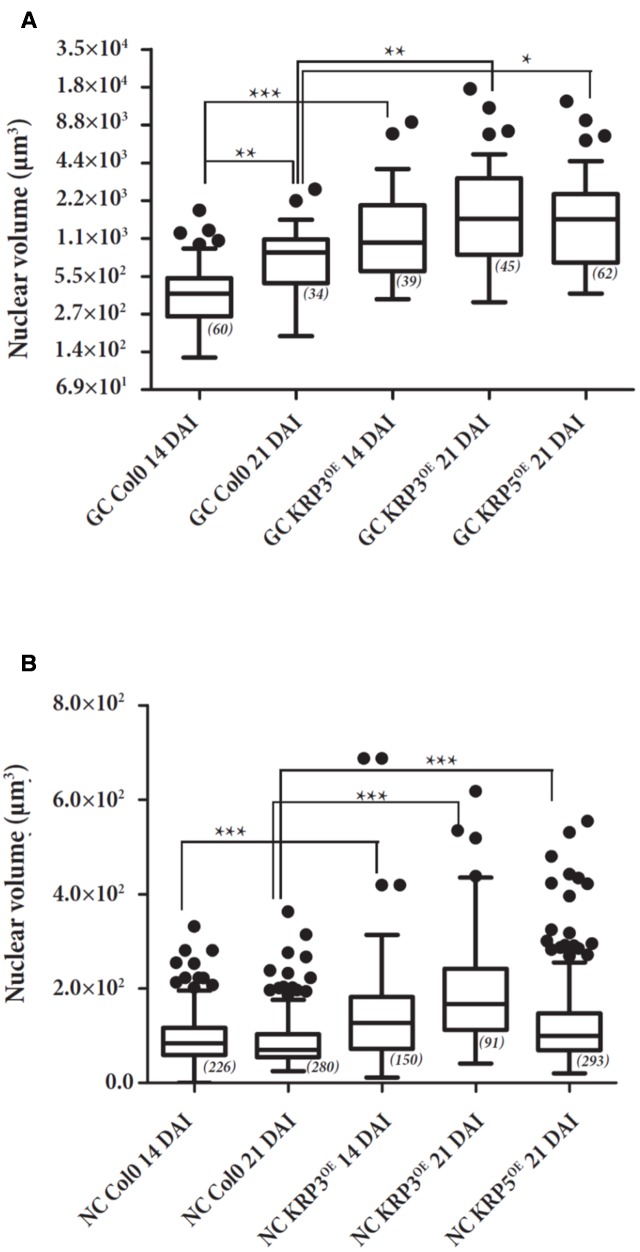
Nuclear volume of GC **(A)** and NGC **(B)** of galls overexpressing KRP3 and KRP5 compared to wild-type. GC and NGC nuclei of *KRP3^OE^* and *KRP5^OE^* and wild-type (Col-0) galls 14 and 21 DAI were measured by the AMIRA imaging software and plotted. Assays were performed in duplicates. Effective is shown between brackets by statistical analysis two-way ANOVA (^∗^, *p* < 0.05), (^∗∗^, *p* < 0.01) and (^∗∗∗^, *p* < 0.001). Error bars represent standard deviation.

**FIGURE 8:**
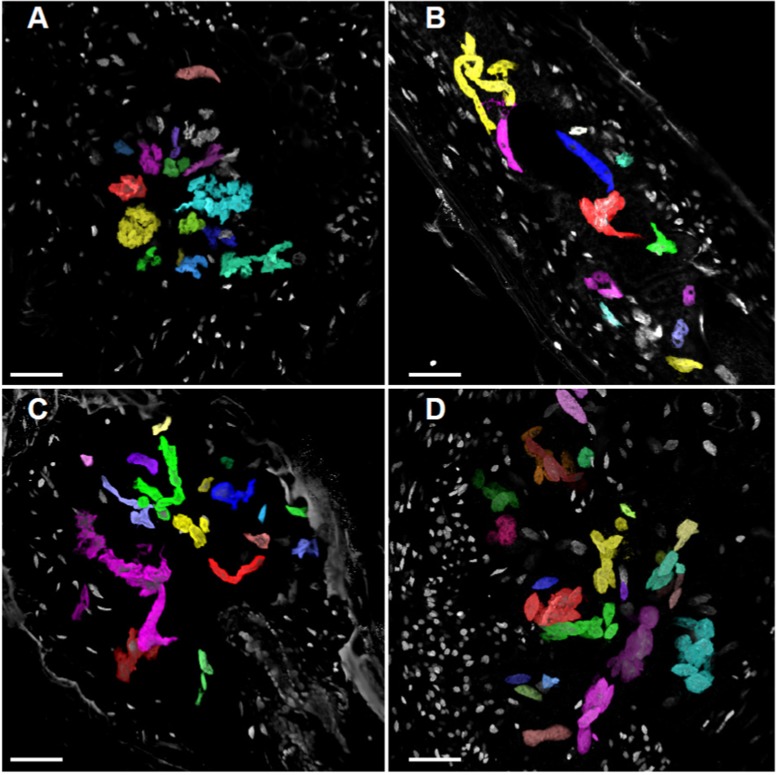
Volume projection of GC and NGC nuclei of wild-type, KRP3^OE^, and KRP5^OE^ galls. Galls in wild-type 21 DAI **(A)**, KRP3^OE^ 14 and 21 DAI **(B,C)** and KRP5^OE^ 21 DAI **(D)** were collected, sectioned and stained with DAPI for confocal imaging of nuclei. Nuclei were segmented and their volume investigated by AMIRA imaging software. GC nuclei are here highlighted with artificial colors. XY maximum intensity projection is featured as background. Bars = 30 μm.

**FIGURE 9:**
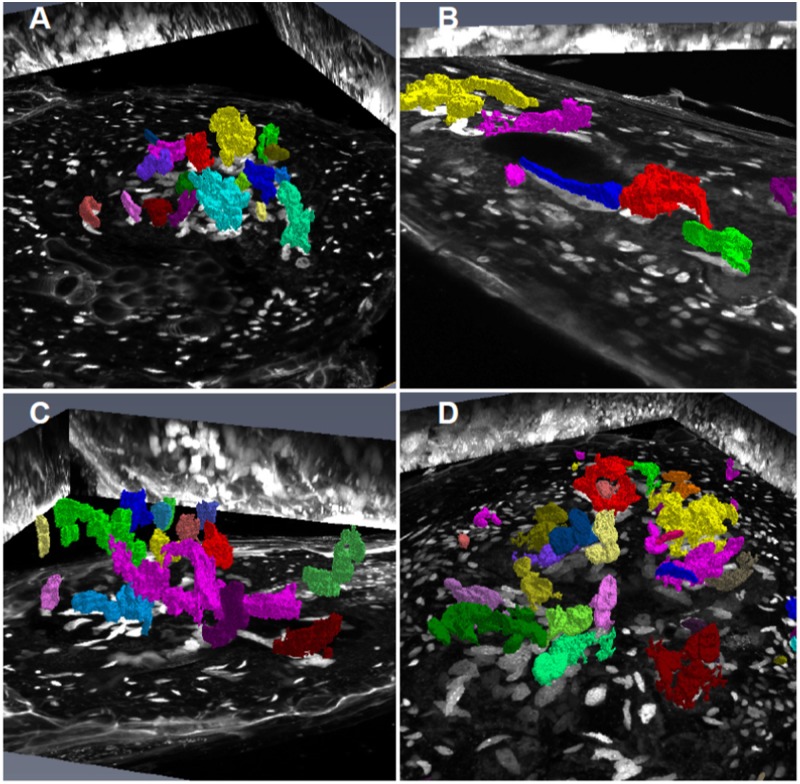
Volume rendering of GC and NGC nuclei from wild-type, KRP3^OE^, and KRP5^OE^ galls. Galls in wild-type 21 DAI **(A)**, KRP3^OE^ 14 and 21 DAI **(B,C)** and KRP5^OE^ 21 DAI **(D)** were collected, sectioned and stained with DAPI for confocal imaging of nuclei. Nuclei were segmented and corresponding volume rendered by AMIRA imaging software. GC nuclei are here highlighted with artificial color. Maximum intensity projections of the three major axes (*xy*, *xz*, and *yz*) are featured as background.

To consolidate the data obtained with the Volumest software, we also analyzed the confocal datasets obtained from the different KRP lines (*KRP3^OE^*, *KRP5^OE^*, and wild-type lines 14 and 21 days after infection with the RKN *M. incognita*) using a 3D analysis dedicated software named AMIRA (**Figure 7**). GC and NGC nuclei were segmented (**Figure 8**) and investigated for their volume (**Figure 9**) via AMIRA imaging software according to the “Materials and Methods” session. Throughout the RKN infection process in the wild-type, we measured a slight but significant (*p* < 0.01) 1.8-fold increase of the GC nuclear volume between 14 and 21 DAI. This volume rise may be explained by the onset of the endoreduplication process. Furthermore, the overexpression of KRP3 at 14 DAI, promotes a 3.8-fold increase of GC nuclear volume compared to wild-type (*p* < 0.001). At 21 DAI, KRP3 and KRP5 overexpression also induced a significant increase in the nuclear volume of approximately threefold compared to wild-type 21 DAI.

No nuclear volume changes of NGC were detected from 14 up to 21 DAI within wild-type galls suggesting the absence of endoreduplication in normally mitotically active NGC. Interestingly, our measurements illustrate that NGC nuclei of galls 14 and 21 DAI for both *KRP3^OE^* and *KRP5^OE^* lines underwent a slight but significant rise of volume 1.45- to 2-fold (*p* < 0.001).

## Discussion

In plant cells, polyploidization is linked with cessation of cell division and initiation of terminal differentiation. Previous data indicate that nematodes maneuver the plant host cell cycle pushing feeding cells to undergo multiple mitotic and endoreduplication cycles resulting in polyploidy. Therefore, deciphering the plant cell cycle machinery in nematode feeding sites will contribute to better understand how nematodes manage to steer the plant cell cycle in their favor.

Classical methods like light- and transmission electron microscopy have been employed to observe nuclei and chromosomes from RKN-induced GCs (Bird, 1961, 1973; Wiggers et al., 1990; Starr, 1993). Confocal microscopy of fixed and *in vivo* specimens allowed us to determine the nuclear 3D shape and to measure nuclear sizes and volumes in nematode-induced GC (Vieira et al., 2012; Coelho et al., 2017). In addition, flow cytometric measurements revealed the high ploidy levels state of GC in our model host *A. thaliana* (Vieira et al., 2012, 2014; Coelho et al., 2017).

Volumetric information can be very helpful when studying individual nuclei with respect to the plant cell cycle in giant-feeding cells induced by RKNs. Ours and other studies have described methodologies to visualize nuclei *in vivo* and/or in fixed GCs in nematode feeding sites using confocal microscopy but none of these studies report GC nuclear volumetric measurements (Vieira et al., 2012, 2013, 2014; Dinh et al., 2014; Hasegawa et al., 2016; Coelho et al., 2017). Different methods have been applied to measure nematode-induced GC area to deduce corresponding volumes (Vieira et al., 2013, 2014; Cabrera et al., 2015) and purely their nuclear area (Vieira et al., 2013, 2014), but there are no reports on GC nuclear volumes. Surface measurements of GC nuclei were reported by Vieira et al. (2013, 2014) using DAPI-stained plastic embedded and sectioned galls. Measuring nuclear surfaces, however, is very labor intensive and strongly influenced by the orientation of nuclei in the section. To avoid tedious histology, Vieira et al. (2012) described a method based on the clearing of whole galls and confocal microscopy imaging. Although very informative, this method is still limited since most galls are covered by multiple layers of neighboring cells (herein named NGC) perturbing an optimal 3D reconstruction of the multiple nuclei dispersed in GCs. We adapted this protocol as outlined in **Figure 2**. Fixed galls were mounted in agarose, sectioned with a vibroslicer, cleared and DAPI stained and finally mounted between two large coverslips while avoiding tissue compression. Thicker samples can, therefore, be imaged at both sides generating high-quality images even for very large galls. Applying the same methodology, galls in other hosts, as well as other complex plant organs for, e.g., in tobacco, rice, soybean, and cotton, can be analyzed. Here, optical sections were generated according to Coelho et al. (2017) by confocal microscopy and image datasets were processed for volumetric measurements using two different softwares: the free Volumest plugin tool from ImageJ and the AMIRA 3D analysis package. These approaches enabled us to compare nuclear volume between galls at different time points, among two *KRP^OE^* transgenic lines compared to wild-type.

The first nuclear volume quantification method we used made use of “Volumest,” an ImageJ plug-in. We measured a user-defined area(s) in a single cross section and, the plug-in generated a three-dimensional volume calculation (Merzin, 2008) based on a series of z-stack images. This plug-in was originally described to be practiced in radiology (Merzin, 2008) and later to measure cell and nuclear volumes of Drosophila brain and epithelium cells (Tamori and Deng, 2013; Doll and Broadie, 2015). Herein, we applied “Volumest” to measure nuclear volumes in nematode-induced GC from wild-type and transgenic Arabidopsis lines overexpressing the cell cycle inhibitors genes *KRP3* and *KRP5*. Since GC nuclei in *KRP3^OE^* and *KRP5^OE^* lines presented peculiar elongated and apparently connected shapes (**Figures 5**, **8**, **9**), we compared their volumes with wild-type GC nuclei. Despite the observed variation in nuclear volume amongst GCs in different lines evaluated, nuclei in *KRP3^OE^* and *KRP5^OE^* lines are consistently larger in volume than in wild-type. These data suggest that defective mitotic events occurring in GC may prevent complete nuclear division and thereby lead to the abnormal increase in nuclear volume. This result is in agreement with the fact that even though there is only a slight increase in ploidy level for the *KRP3^OE^* line, a considerable increase in nuclear volume is observed. Moreover, despite the vast increase in GC nuclear volume in the *KRP5^OE^* line, previous flow cytometry data showed no increase in ploidy levels for this line supporting the idea that the nuclear volume increase observed for these mutants is not caused by an increase in DNA content but rather a consequence of mitotic defects (Coelho et al., 2017). Using the “Volumest” method we were unable to observe minor differences in nuclear volumes of NGC for the three different lines.

As a second measuring approach we used AMIRA, a commercial software package which is based on morphological segmentation of the image data set. This segmentation relies on a white top-hat transformation to extract objects (here nuclei) from the data set. The size of the extracted object is conditioned by a self-chosen kernel size for a mathematical operation called “opening.” In digital image processing, an opening is the dilation of the erosion of the image by a structuring element named a kernel matrix. While opening removes small objects from the foreground, it can also be used to extract specific features from the image that fits the kernel matrix. Segmented objects are finally properly labeled and analyzed for volumetrics. Since the latter method involves image processing before measuring, it is expected to be less depending on manual errors.

Interestingly, measurements by AMIRA revealed that GCs nuclei in galls 21 DAI in the *KRP3^OE^* and *KRP5^O^*^E^ lines increased up to three times in volume compared to wild-type GC nuclei. A slight increase of NGC nuclear volume was also observed for both *KRP* lines within a range of 1.5–2 times for *KRP3^OE^* and *KRP5^OE^* 21 DAI as compared to wild-type NGC nuclei. These results suggest that ectopic KRP3 and KRP5 expression in galls promotes an increase in the nuclear volume of GC and to a lesser extent but also true for NGC nuclei.

The results obtained from both volumetric measurement methods show that giant cell nuclei are significantly bigger in both *KRP3^OE^* and *KRP5^OE^* lines than in the wild-type. These data together with volume rendering of giant cell nuclei suggest that *KRP3^OE^* and *KRP5^OE^* lines accumulate mitotic defects which can lead to disturbed mitotic events and erroneous nuclear division. These defects may explain the elongated and undulated nuclear phenotype observed in GC of both KRP overexpressing lines (seen in **Figure 5**). Despite that both analysis point in the same direction, nuclear volume differences are more pronounced when using the AMIRA approach. The latter also enabled to detect minor but significant volumetric variation when comparing nuclei from different plant lines. Several criteria may explain such differences: a lack of reproducibility when using Volumest which relies on the manual delineation of each nucleus determined by the user, and its limited ability to discriminate all shades of gray preventing the accurate delineation of nuclear boundaries. This error may affect the volume output and the observed differences between the two methods. Besides, the highly irregular nuclear shapes observed in the KRP lines may hamper the selection of a single nucleus which actually might consist of two entangled entities in very close proximity. A manual measuring procedure will be more prone to introducing this type of error. Since Volumest seems less accurate, it may also have prevented to observe the increase of NGC nuclear volume in KRP3*^OE^* and KRP5*^OE^* lines during the nematode infection process.

Although our analysis revealed that the Volumest approach seems to provide somewhat less accurate results than AMIRA, the former still features some advantages: -it is a free public domain software, -there is no need for additional mathematical operation and, -it is easy in use relying on hand-made object delineation. Since “Volumest” is supported by ImageJ, it is up to the user to implement additional data processing tools such as the “top-hat” approach prior to “Volumest” analysis.

It is important to mention that recently Ohtsu et al. (2017) have described a method for imaging CN-induced syncytia using the ClearSee clearing method (Kurihara et al., 2015). In this work, the authors have used the software IMARIS (Bitplane, United Kingdom) to assess syncytia nuclear volume. The authors were able to nicely image whole syncytia samples of *Astragalus sinicus* roots infected with *Heterodera glycines*. The strategy we propose here and which is based on root vibroslicing combined with nuclear volume measurement (ImageJ and AMIRA) can also be applied for much thicker roots infected with cyst or RKN. Currently, thick vibrosliced samples are being optimized using different clearing approaches for deep tissue imaging.

Summing up, “Volumest” ImageJ and AMIRA softwares have been applied to determine nuclear volumes of GC and NGC nuclei from transgenic lines overexpressing *KRP3* and *KRP5* compared to wild-type. Both lines presented exceptionally elongated and apparently connected nuclear morphology. These phenotypes are characteristically occurring in some KRP overexpressing lines and were enhanced in mature GC (21 DAI) compared to young feeding cells (5–10 DAI), most likely due to cumulative defective mitotic events (de Almeida Engler et al., 2012; Vieira et al., 2012). Remarkably, differences observed in nuclear ploidy and volume in GC show that an increase in nuclear volume is not necessarily linked to ploidy levels. The evident accumulation of mitotic defects in GC nuclei upon overexpression of *KRP3* and *KRP5* could to a certain degree prevent optimal cell cycle progression. The two methods used here for volumetric measurements provide valuable information that complements former data obtained by flow cytometry. Previous records combined with the results obtained here suggest that the increase in nuclear volume, as well as the formation of highly extended and undulated nuclei in GCs overexpressing KRP3 and KRP5, may largely result from mitotic defects such as incomplete chromosomes separation rather than endoreduplication.

## Author Contributions

JDASJ, OP, GE, and JdA have conceived and designed the paper; JDASJ, OP, and RC have performed the experiments; JDASJ, OP, RC, JdA, and GE have analyzed the data; MG-d-S, GE, and JdA have contributed with reagents/materials/analysis tools; JDASJ, JdA, and GE have written the paper; MG-d-S, OP, GE, and JdA, have amended the paper (helped writing legends, M&M, added suggestions).

## Conflict of Interest Statement

The authors declare that the research was conducted in the absence of any commercial or financial relationships that could be construed as a potential conflict of interest.

## Footnotes

1 http://lepo.it.da.ut.ee/~markkom/volumest/
