# Supplementary material for: Application of Nuclear Volume Measurements to Comprehend the Cell Cycle in Root-Knot Nematode-Induced Giant Cells
Source: Front Plant Sci. 2017 Jun 12;8:961. doi: 10.3389/fpls.2017.00961 (PMC5466992; doi:10.3389/fpls.2017.00961)
Supplement: Supplementary file 1 [file Presentation_1.PDF]

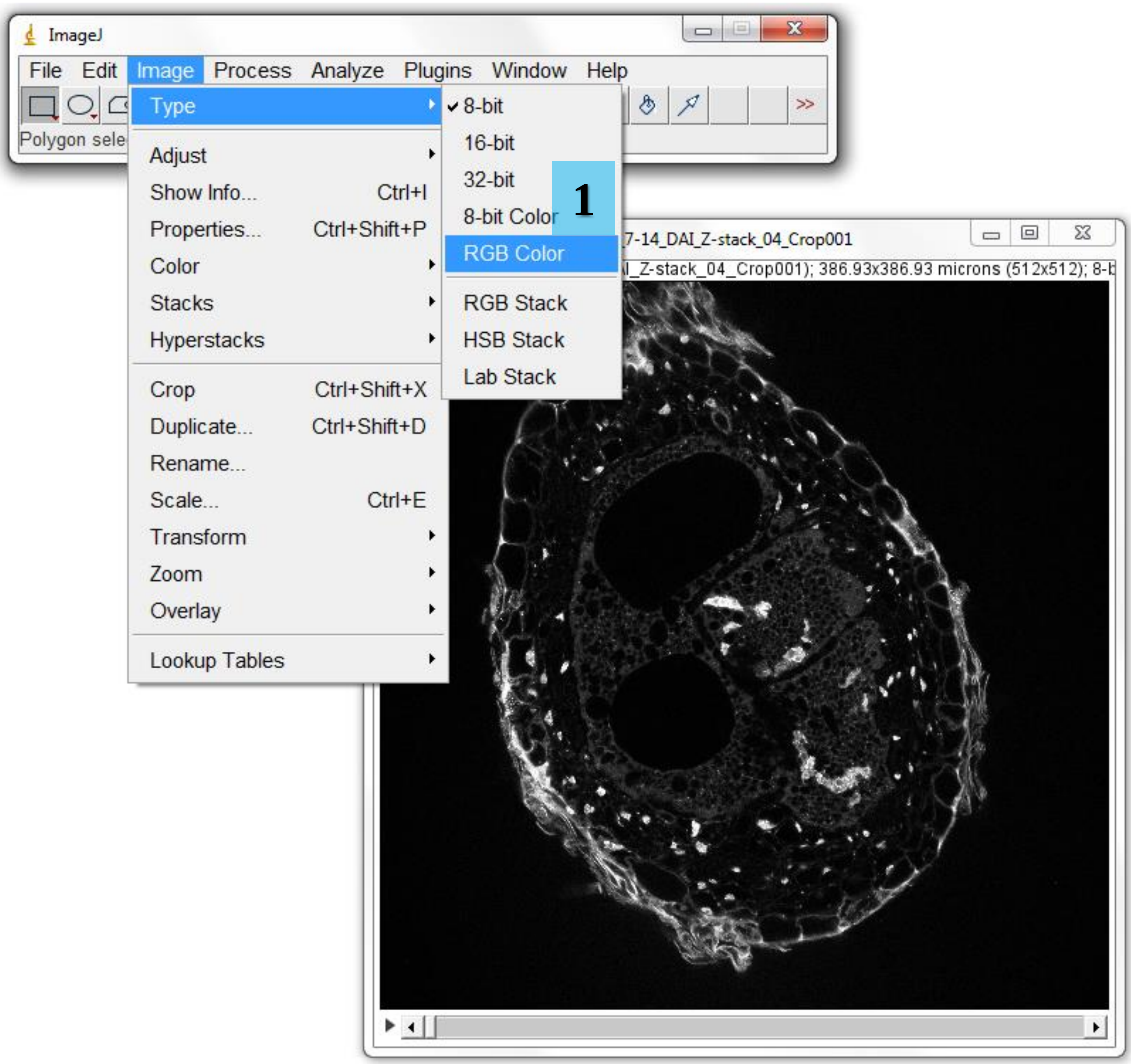

Measuring gall nuclei from confocal sections using the Image J plugin, Volumest.

Download the Image J plug-ins: LOCI, that allows Image J to open any confocal output format, and Volumest. The Volumest plugin can be found at http: //lepo.it.da.ut.ee/~markkom/volumest/

1.Once the confocal Z-stack is opened, it has to be transformed to RGB color, like indicated in the image.

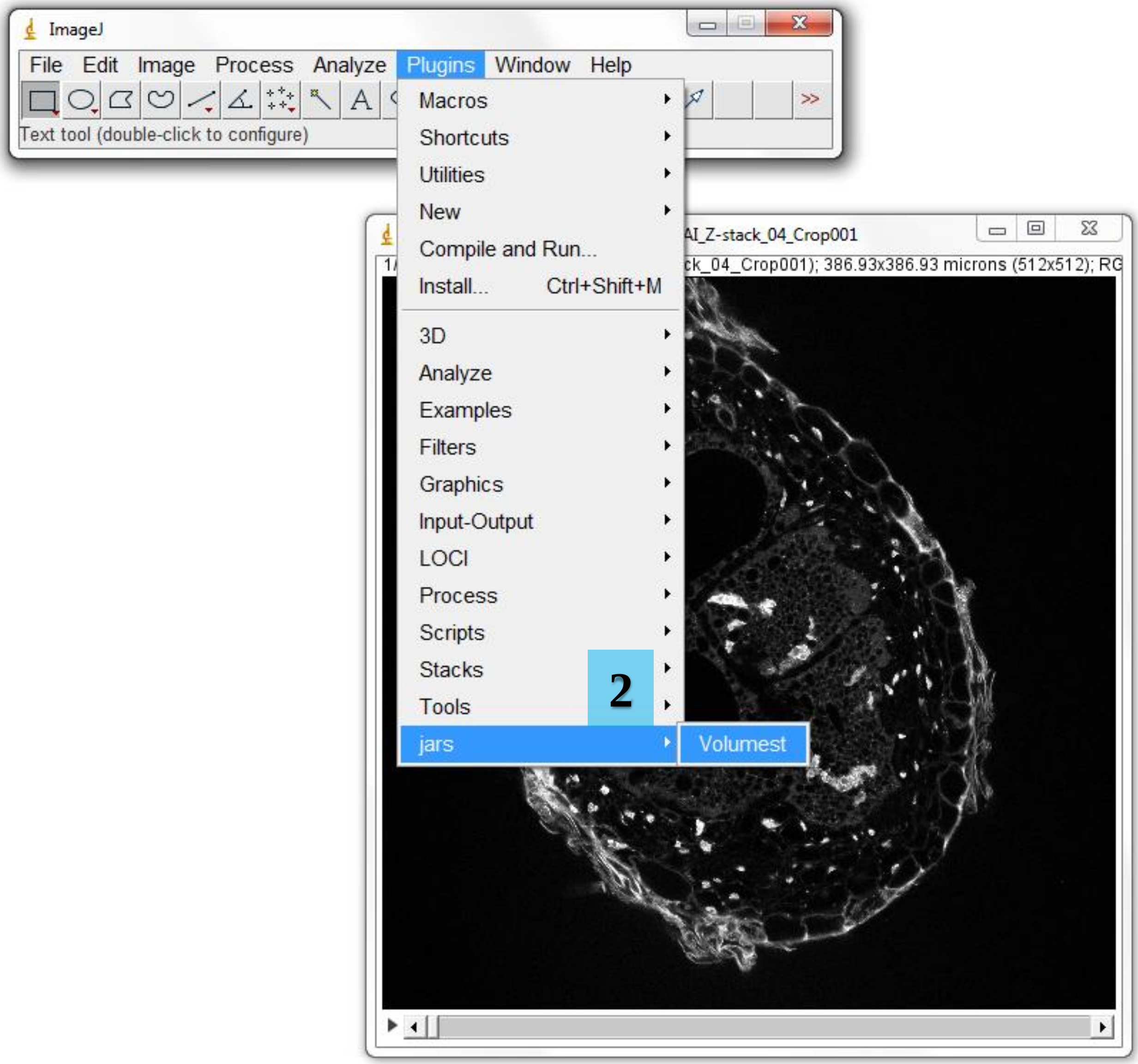

2. Open Volumest as shown above.

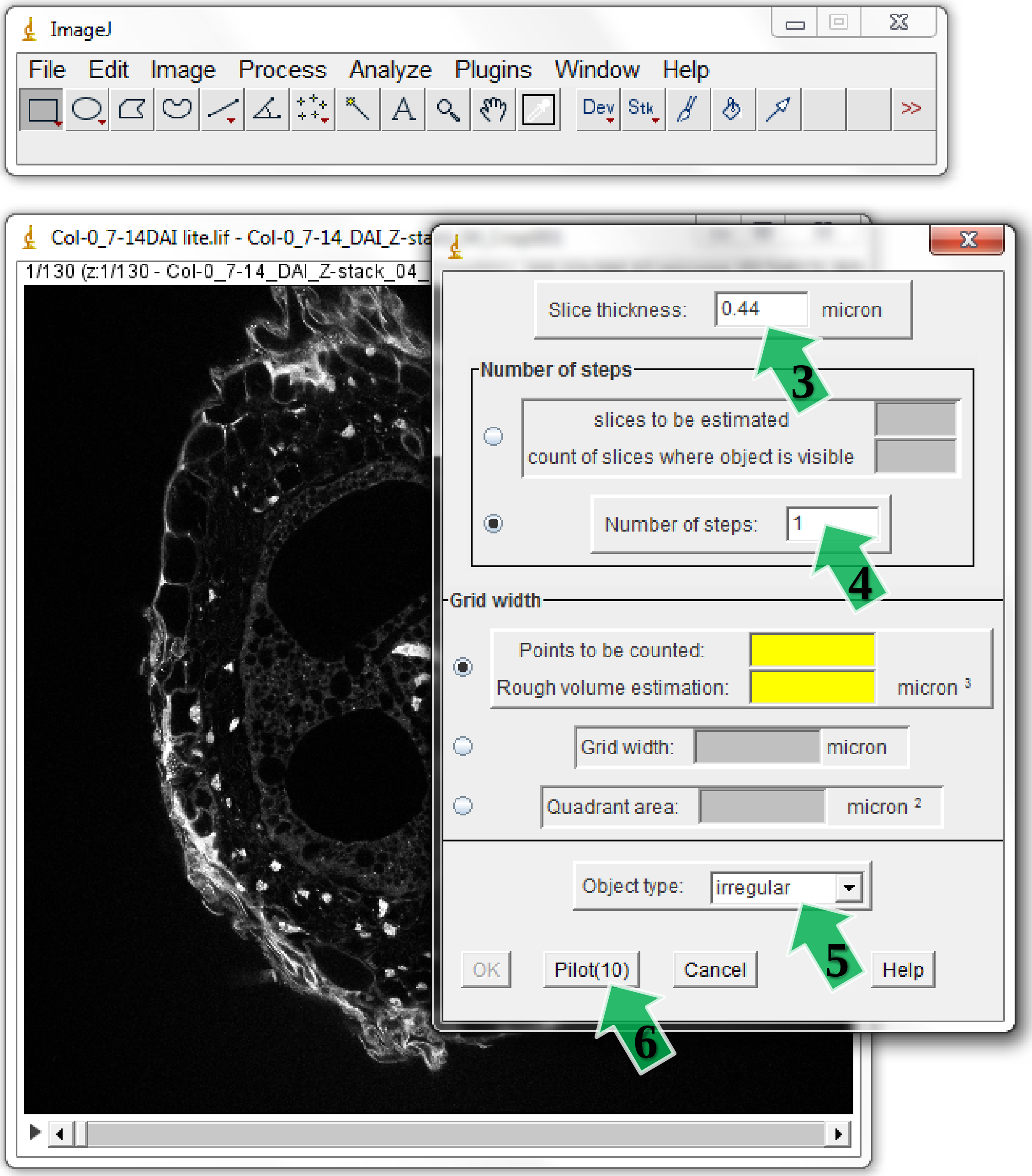

3. Set the slice thickness. This information should be accordingly the confocal image settings

4. Set the number of steps for measuring. Here, we have tested the number of steps from 1 to 5 without loosing confidence in measuring. Although, for other types of samples it should be tested again.

5. Set the “object type” as **irregular**

6. Press “Pilot” to allow setting the grid width

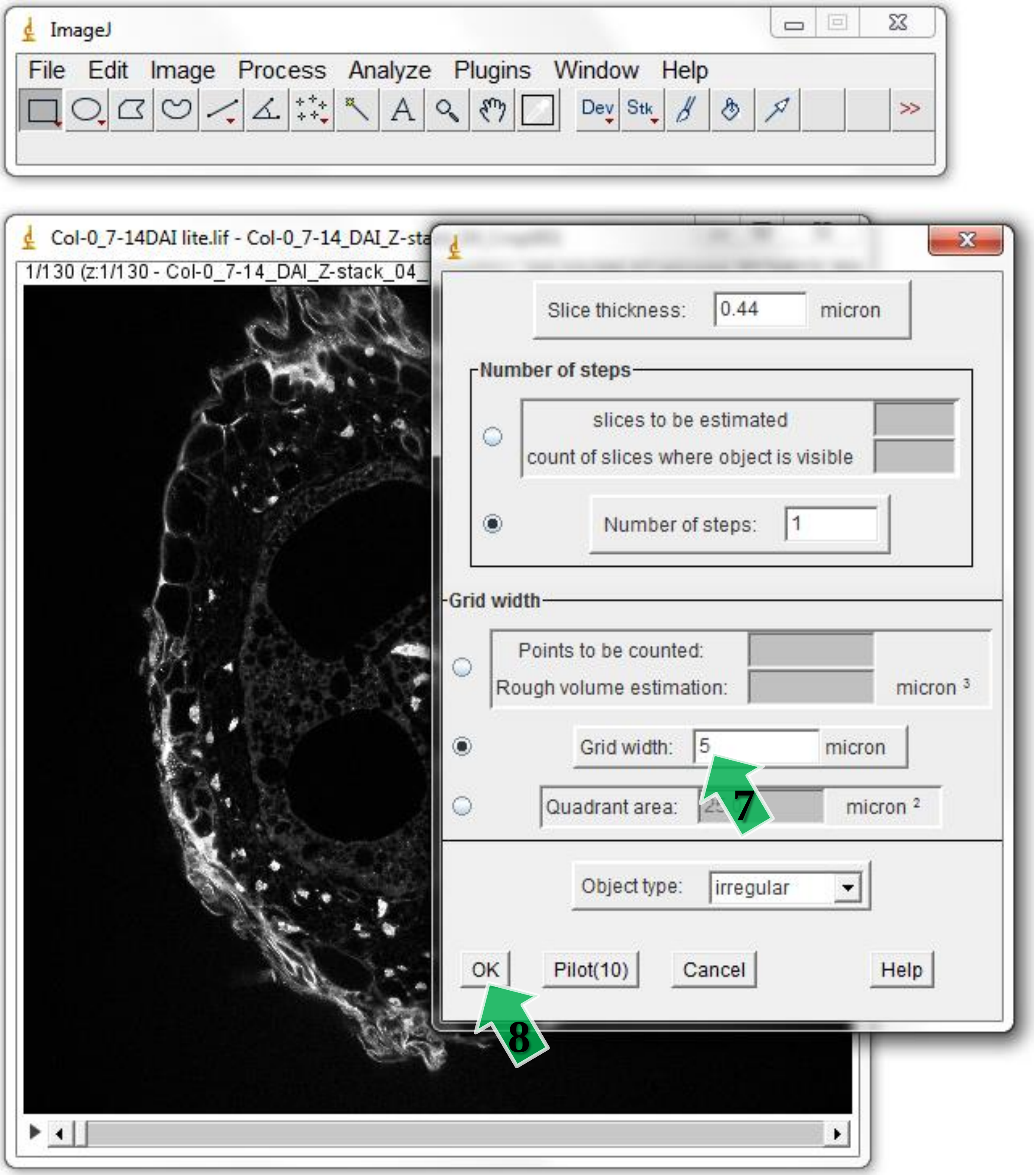

7. Set the grid width to 5 micron. In our work 25 square microns were suitable for gall nuclei imaged with the 40X immersion objective lens.

8. Press “OK”

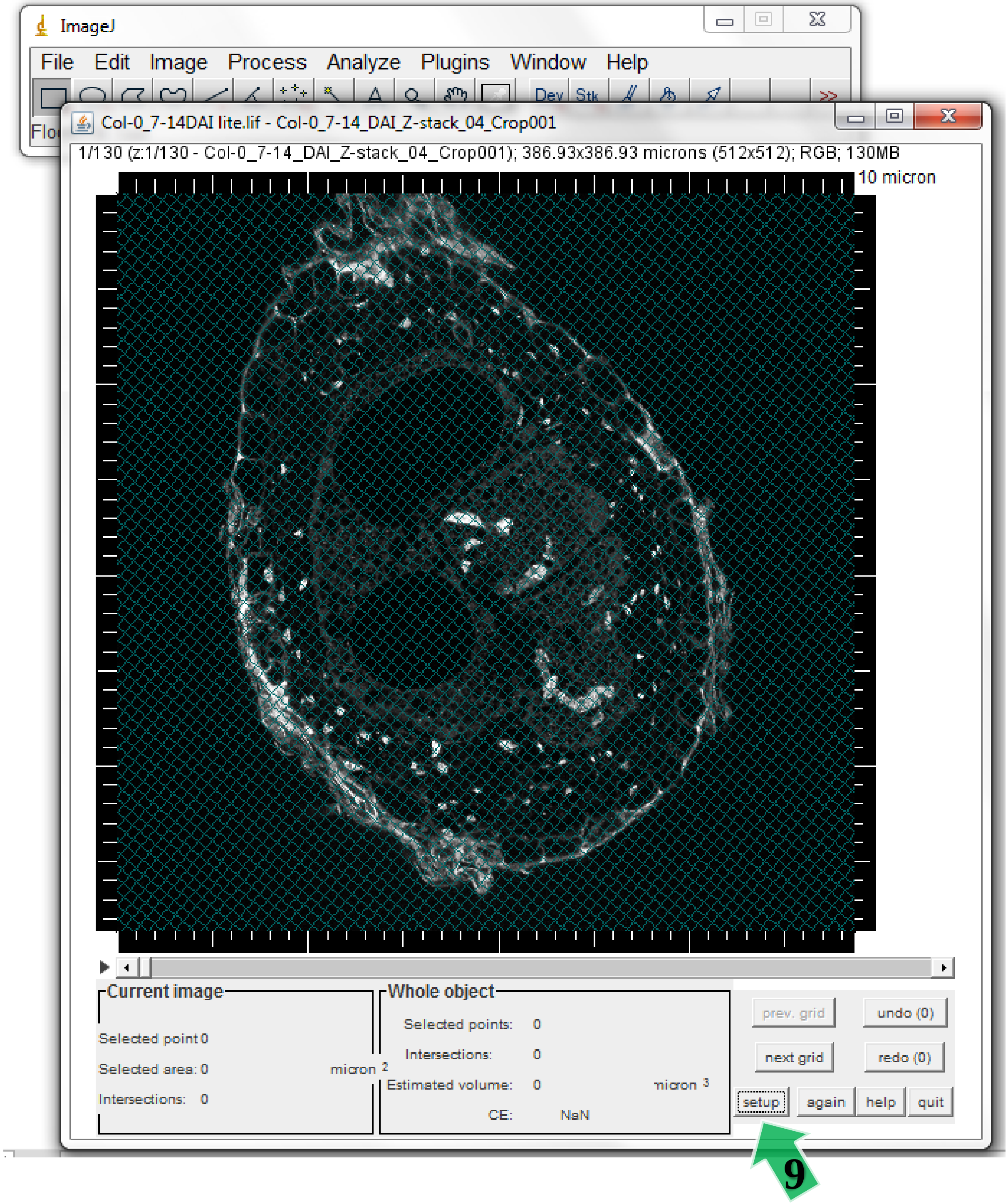

9. Push “setup” to change the grid color to achieve better contrast avoiding interference while measuring.

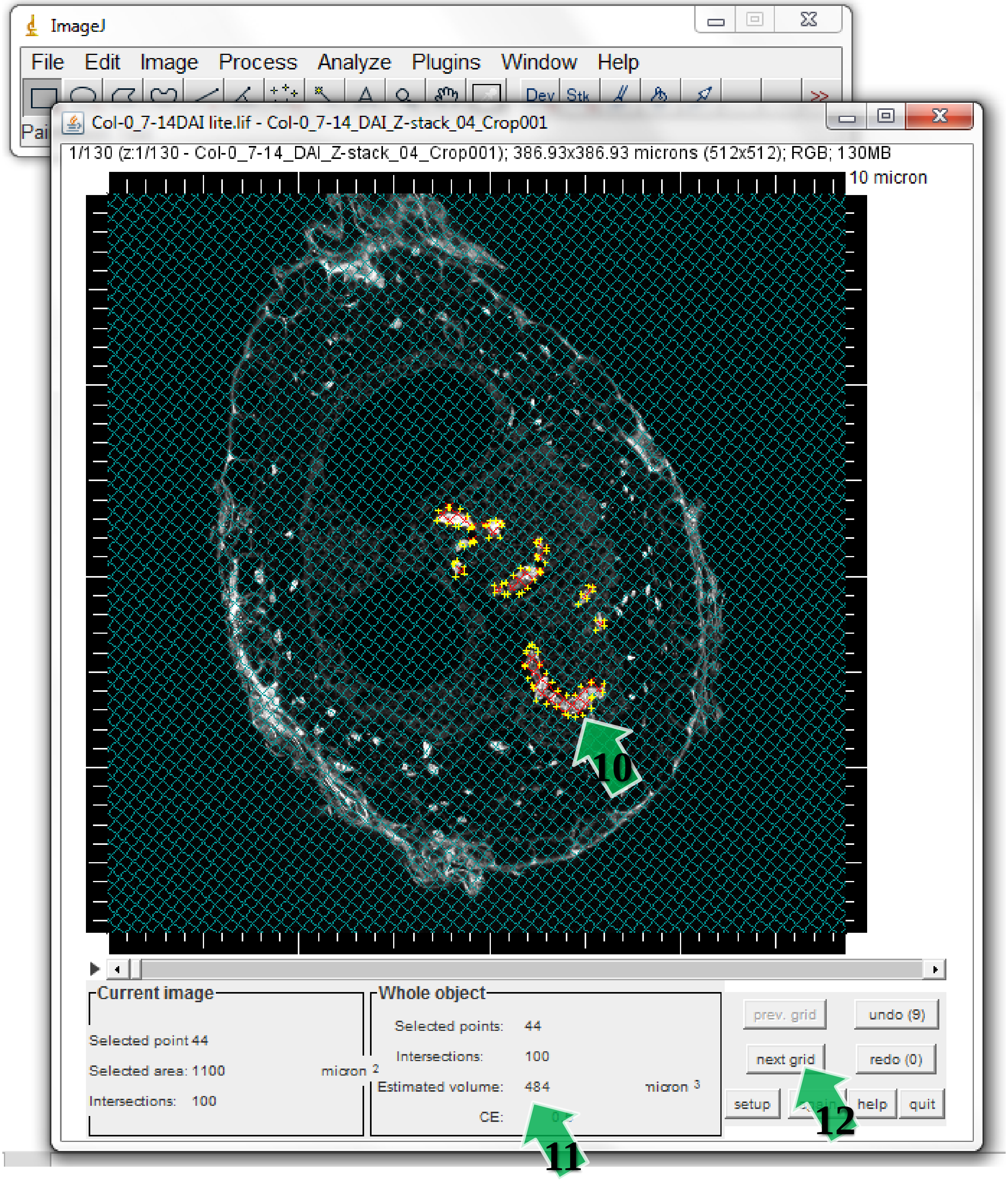

10. Measuring is done by pressing and holding the mouse arrow while moving around the desired objects , in this case, the DAPI stained nuclei.

11. The cumulative volume can be followed at the square named “whole object”. It is possible to measure individual nuclei or all together.

12. When measuring the first optical slice is finished, press “next grid” to measure the next slice of the confocal Z-stack. To finalize, record the cumulated volume at “estimated volume” (see step 11).
